# Characterization of Phospho-(Tyrosine)-Mimetic Calmodulin Mutants

**DOI:** 10.1371/journal.pone.0120798

**Published:** 2015-04-01

**Authors:** Silviya R. Stateva, Valentina Salas, Gustavo Benaim, Margarita Menéndez, Dolores Solís, Antonio Villalobo

**Affiliations:** 1 Instituto de Investigaciones Biomédicas, Consejo Superior de Investigaciones Científicas and Universidad Autónoma de Madrid, Madrid, Spain; 2 Universidad Central de Venezuela, Facultad de Ciencias, Instituto de Biología Experimental, Caracas, Venezuela; 3 Instituto de Estudios Avanzados (IDEA), Caracas, Venezuela; 4 Instituto de Química Física Rocasolano, Consejo Superior de Investigaciones Científicas, Madrid, Spain; The University of Tokyo, JAPAN

## Abstract

Calmodulin (CaM) phosphorylated at different serine/threonine and tyrosine residues is known to exert differential regulatory effects on a variety of CaM-binding enzymes as compared to non-phosphorylated CaM. In this report we describe the preparation and characterization of a series of phospho-(Y)-mimetic CaM mutants in which either one or the two tyrosine residues present in CaM (Y99 and Y138) were substituted to aspartic acid or glutamic acid. It was expected that the negative charge of the respective carboxyl group of these amino acids mimics the negative charge of phosphate and reproduce the effects that distinct phospho-(Y)-CaM species may have on target proteins. We describe some physicochemical properties of these CaM mutants as compared to wild type CaM, after their expression in *Escherichia coli* and purification to homogeneity, including: i) changes in their electrophoretic mobility in the absence and presence of Ca^2+^; ii) ultraviolet (UV) light absorption spectra, far- and near-UV circular dichroism data; iii) thermal stability in the absence and presence of Ca^2+^; and iv) Tb^3+^-emitted fluorescence upon tyrosine excitation. We also describe some biochemical properties of these CaM mutants, such as their differential phosphorylation by the tyrosine kinase c-Src, and their action as compared to wild type CaM, on the activity of two CaM-dependent enzymes: cyclic nucleotide phosphodiesterase 1 (PDE1) and endothelial nitric oxide synthase (eNOS) assayed *in vitro*.

## Introduction

Calmodulin (CaM) is a highly conserved Ca^2+^-receptor protein, ubiquitous in all eukaryotic organisms, implicated in the regulation of many cellular systems by transducing changes in the concentration of free Ca^2+^ in the cytosol and other intracellular compartments and structures (reviewed in [1]). CaM is multifunctional and controls a myriad of cellular processes ranging from egg fertilization, muscle contraction, osmotic control and ions transport, metabolism, gene expression, cell migration, proliferation, autophagy and programmed cell death, among many others, and plays a significant role in functions that are deregulated in pathological processes such as cancer (reviewed in [2]), arrhythmias and infant cardiac arrest [3, 4], and other ailments such asthma or chronic obstructive pulmonary disease (reviewed in [5]) among others. By interacting with close to three hundred target proteins, with and without enzymatic activity, and regulating their functions in a temporo-spatial fashion, CaM achieves this feast. Distinct mechanisms of interaction between the Ca^2+^/CaM complex and CaM-binding proteins have been described [6, 7]. In addition to the Ca^2+^-mediated modulation of CaM action, apocalmodulin (Ca^2+^-free CaM) also interacts with and regulates the activity of a variety of CaM-binding proteins [8, 9].

An assortment of serine/threonine- and tyrosine-protein kinases have been shown to phosphorylate CaM *in vitro* and in living cells. The distinct phospho-CaM species differentially regulate the activity of many target proteins as compared to non-phosphorylated CaM (reviewed in [10]). This suggests that this posttranslational modification could play additional and/or complementary roles to Ca^2+^ binding in the modulation of CaM activity.

The non-receptor tyrosine kinase Src [11], the insulin receptor [12], and the epidermal growth factor receptor (EGFR) [13], are prominent kinases implicated in the phosphorylation of CaM at both Y99 and Y138, the only two tyrosine residues present in vertebrate CaM. It seems likely that the dynamic identification of distinct phosphorylated forms of CaM at a given time acting on specific targets could be difficult to observe in living cells due in part to the short half-life of phospho-(Y)-CaM. An alternative to explore these events could be to use recombinant phospho-(Y)-mimetic CaM mutants.

In order to understand the implication of distinct phospho-(Y)-CaM species on the activity of different CaM-binding target proteins, we have prepared a set of recombinant phospho-(Y)-mimetic CaM mutants by substituting either one or the two tyrosine residues to aspartic acid or glutamic acid. Here we characterized their physico-chemical properties and tested *in vitro* their action on the activity of two CaM-dependent enzymes: the 3’,5’-cyclic nucleotide phosphodiesterase 1 (CaM-dependent isoform) (PDE1) (EC 3.1.4.17) and endothelial nitric oxide synthase (eNOS) (EC 1.14.13.39).

## Materials and Methods

### Reagents

Phenyl-Sepharose 6 (fast flow), (6R)-5,6,7,8-tetrahydrobiopterin dihydrochloride, FMN, FAD, cAMP, 3’,5’-cyclic nucleotide phosphodiesterase 1 (CaM-dependent isoform) (PDE1) (from bovine brain), 5’-nucleotidase (EC 3.1.3.5) (from *Crotalus atrox* venom), eNOS (bovine recombinant baculovirus-expressed in Sf9 cells), histone (type II-A), and anti-mouse IgG (Fc specific) peroxidase-conjugated secondary antibody were obtained from Sigma-Aldrich Co. (St. Louis, MO). The ultrasensitive colorimetric assay kit for eNOS was purchased from Oxford Biomedical Research (Rochester Hills, MI). Rabbit monoclonal anti-Src antibody (clone 36D10, isotype IgG) was purchased from Cell Signaling Technology Inc. (Danvers, MA). Mouse monoclonal anti-phosphotyrosine antibody (clone 4G10, isotype IgG_2bκ_), recombinant c-Src (human) and mouse monoclonal anti-CaM antibody (isotype IgG_1_) were obtained from Millipore (Billerica, MA). Anti-rabbit IgG horseradish peroxidase-linked secondary antibody was purchased from Invitrogen (Eugene, OR). The QuickChange XL site-directed mutagenesis kit was acquired from Agilent Technology (Santa Clara, CA), and the QIAprep plasmid preparation kit from Quiagen Ltd. (Manchester, UK). The Slide-A-Lyzer dialysis cassettes were purchased from Thermo Scientific-Pierce (Rockford, IL). Competent *Escherichia coli* BL21(DE3)pLysS was purchased from Stratagene (La Jolla, CA), and the BugBuster protein extraction reagent kit was from Merck/Millipore (Darmstadt, Germany). The pETCM vector was kindly provided by Prof. Nobuhiro Hayashi from the Institute for Comprehensive Medical Science, Fujita Health University, Japan.

### Generation of calmodulin mutants

Polymerase chain reaction-aided site-directed mutagenesis was performed in the pETCM vector containing the coding sequence of *Rattus norvegicus* CaM gene II [14] as template using the QuickChange XL Site-Directed Mutagenesis kit and the following set of complementary oligos: 5’-GGCAATGGCGACATCAGTGCAGCA-3’ and 5’-TGCTGCACTGATGTCGCCATTGCC-3’ for the Y99D substitution; 5’-GGGGATGGTCAGGTAAACGACGAAGAGTTTGTACAAATG-3’ and 5’-CATTTGTACAAACTCTTCGTCGTCGTTTACCTGACCATCCCC-3’ for the Y138D substitution; 5’-GGCAATGGCGAGATCAGTGCAG-3’ and 5’CTGCACTGATCTCGCCATTGCC-3’ for the Y99E substitution; and 5’-GGGGATGGTCAGGTAAACGAGGAAGAGTTTGTACAAATG-3’ and 5’-CATTTGTACAAACTCTTCCTCGTCGTTTACCTGACCATCCCC-3’ for the Y138E substitution. Sequential mutagenesis was carried out to obtain the double substitutions Y99D/Y138D and Y99E/Y138E using the Y99D and Y99E mutated vectors, respectively, as template. *Escherichia coli* DH5α was transformed with the pETCM, pETCM(Y99D), pETCM(Y138D), pETCM(Y99D/Y138D), pETCM(Y99E), pETCM(Y138E) or pETCM(Y99E/Y138E) vectors for their replication, and purification by the alkaline lysis method [15] using the QIAprep plasmid preparation kit. The correctness of the mutagenesis procedures was ascertained by sequencing the vectors using an oligo annealing to the T7 promoter.

### Expression and purification of recombinant calmodulin mutants

*E*. *coli* BL21(DE3)pLysS was transformed with the vectors indicated above using a thermic-shock protocol. Single colonies grown in solid medium in the presence of 100 μg/ml ampicillin were collected and seeded in 5 ml of Luria´s broth containing the same concentration of ampicillin. Larger cultures (500 ml) were seeded with the pre-culture and grown in the same conditions until they reached an OD_600nm_ = 0.7 ± 0.1. The expression of the recombinant proteins was induced with 0.5 mM isopropyl-1-thio-β-D-galactopyranoside (IPTG) during 4 hours at 37°C. Control cultures in the absence of IPTG were included. The bacteria were collected by centrifugation at 6,000 x g for 20 min and frozen at -70°C until used. The bacteria were lysed using the BugBuster protein extraction reagent kit, centrifuged at 11,000 x g during 10 min at 4°C and the supernatant collected, heated at 95°C for 5 min and centrifuged again as above. Heat-resistant CaM present in the new supernatant was purified as previously described [14]. Briefly, phenyl-Sepharose 6 Fast Flow was prepacked in a column and equilibrated with a buffer containing 50 mM 2-amino-2-hydroxymethyl-propane-1,3-diol (Tris)-HCl (pH 7.5) and 1 mM CaCl_2_. The sample was applied to the column and washed with four column volumes of a buffer containing 50 mM Tris-HCl (pH 7.5), 1 mM CaCl_2_ and 100 mM NaCl. CaM was eluted with a buffer containing 50 mM Tris-HCl (pH 7.5) and 2 mM EGTA in fractions of 10 ml. The fractions containing CaM were desalted using Slide-A-Lyzer dialysis cassettes with membranes of 3.5 kDa cut-off against a buffer containing 10 mM 4-(2-hydroxyethyl)-1-piperazineethanesulfonic acid (Hepes)-NaOH (pH 7.5) or 20 mM Tris-HCl (pH 7.5) and kept at a concentration of 1–2 mg/ml at -70°C. Protein concentration was determined by the bicinchoninic acid method [16] using bovine serum albumin as standard. The purity of the samples was confirmed by polyacrylamide gel electrophoresis in the presence of sodium dodecyl sulfate (SDS-PAGE) [17] in a 5–20% (w/v) continuous gradient slab gel adding 5 mM EGTA or 1 mM CaCl_2_ in the loading buffer to attain the characteristic Ca^2+^-induced electrophoretic mobility shift of CaM [18], and staining the gel with Coomassie Brilliant Blue R-250.

### Absorption spectra

The absorption spectra in the range 240–340 nm of the different species of CaM (120 μM) were measured in 20 mM Tris-HCl (pH 7.5) using a CARY/1E/UV-Visible spectrophotometer (Varian) equipped with Cary winUV software.

### Circular dichroism

Far-UV (200–260 nm) and near-UV (250–320 nm) circular dichroism spectra of the different CaM species were measured at 20°C in the presence of either 1 mM EGTA or 1 mM CaCl_2_ in a buffer containing 20 mM Tris-HCl (pH 7.5) and 0.1 M KCl using a JASCO J-810 spectropolarimeter (Cremelia, Italy) equipped with a Peltier type temperature control system (Model PTC-423S/L), a bandwidth of 0.2 nm and a response time of 4 s. Far-UV spectra were recorded in 0.1 cm path-length quartz cells at a protein concentration of 0.2 mg/ml, and near-UV spectra in 1 cm path-length cuvettes at a protein concentration of 2 mg/ml as previously described [19]. The Spectra Manager software (JASCO, v 1.52.01) was used for data collection and analysis. Routinely, the corresponding buffer baseline was subtracted and the corrected data were normalized per mole of amino acid. Thermal denaturation experiments were carried out in the same system by increasing the temperature from 20 to 90°C at a scanning rate of 0.66°C/min. Variations in ellipticity at 222 nm were monitored at steps of 0.2°C.

### Tb^3+^ fluorescence

The emission fluorescence spectra in the range 520–580 nm of the different species of CaM (10 μM) upon excitation of tyrosine (if present) at 280 nm were measured in 10 mM 1,4-piperazinediethanesulfonic acid (Pipes)-HCl (pH 6.5), 100 mM KCl, and increasing concentrations of TbCl_3_ (0–200 μM) using a Photon Technology International Inc. spectrofluorometer system (Birmingham, NJ). The Tb^3+^ emission fluorescence peak at 543 nm upon tyrosine excitation at 280 nm was quantified as previously described [20].

### Isothermal titration calorimetry (ITC)

Determination of the kinetics parameters of PDE1 in the absence and presence of wild type CaM and CaM(Y99D/Y138D) were done in a VP-ITC instrument (GE-Healthcare). The reaction cell (1.4619 ml) was filled with degassed protein solutions and equilibrated at 37°C. Stirring speed was 307 rpm and thermal power was registered every 2 seconds with an instrumental reference power of 20 μcal/s. Enzyme reaction rates were determined by measuring the change in the instrumental power supplied to the sample cell after addition of the substrate [21]. At least three independent measurements were carried out in each experimental condition. Serial injections of 4.7 mM cAMP were made every 2 min into the sample cell loaded with (0.01–0.04 U) phosphodiesterase solutions containing or not either wild type CaM or the Y99D/Y138D mutant (3.8 μM). To avoid distortions associated with dilution events, the power change associated with substrate addition at injection *i* was averaged over the 30 s immediately before the injection *i*+1. Reaction rates under steady-state conditions were then calculated dividing the power change for each injection, (*dQ*/*dt*)_*i*_, by the cell volume and the apparent reaction enthalpy (Δ*H*_*app*_) using the software provided by the manufacturer (Origin ITC 7.0). The complete enthalpy of reaction was determined in separate experiments where 15 μl of 4.7 mM cAMP were injected into the cell filled with a solution of (0.1 units) PDE1 and 3.8 μM CaM wild type and the hydrolysis was monitored until position of the initial base was recovered. Integration of the area under the peak gave the total heat of hydrolysis that was corrected by the heat of cAMP dilution, measured independently by injecting the substrate solution in the sample cell loaded with buffer. Very similar values of Δ*H*_*app*_ (-19.9 ± 0.5 kcal/mol) were obtained with subsequent injections, indicative of no product inhibition.

### Phosphorylation of calmodulin

Phosphorylation of CaM was carried out at 37°C for 30 min in a total volume of 0.1 ml in a buffer containing 15 mM Hepes-NaOH (pH 7.4), 5 mM MgCl_2_, 1 mM EGTA, 1 mM dithiothreitol, 1.2 μM histone, 1.2 μM CaM (different species), and 0.02 μg of recombinant c-Src. The reaction was started upon addition of 2 mM ATP and stopped upon addition of Laemmli sample buffer and heating the sample at 100°C for 5 min. Tyrosine-phosphorylated CaM and auto-phosphorylated c-Src were detected by standard Western blot technique using the anti-phospho-tyrosine 4G10 antibody. Membrane segments were stripped at 50°C for 45 min in a buffer containing 2% (w/v) SDS, 60 mM Tris-HCl (pH 6.8) and 0.1% (v/v) β-mercaptoethanol, extensively washed with water, incubated in TBST buffer (0.1% (w/v) Tween-20, 100 mM Tris-HCl (pH 8.8), 500 mM NaCl and 0.25 mM KCl) for 30 min and reprobed with either anti-Src or anti-CaM antibodies as loading controls. Alternatively, the phosphorylation assay was performed in 0.1 ml of medium containing 15 mM Hepes–NaOH (pH 7.4), 5 mM MgCl_2_, 1 mM EGTA, 1 mM dithiothreitol, 1.2 μM histone, 1.2 μM CaM, 10 μM (2 μCi) [γ-^32^P]ATP, and 2 units c-Src at 37°C for 30 min, and detection of ^32^P-labeled phospho-(Y)-CaM was done by autoradiography as previously described [22]. One unit of c-Src activity corresponds to the incorporation of 1 nmol of phosphate into 250 μM cdc2 substrate peptide per min at 30°C using 100 μM ATP according to the manufacture’s datasheet. When larger amounts of phospho-(Y)-CaM was required to assay its action on target proteins, CaM (1 mg) was phosphorylated in a 1 ml reaction mixture containing 400 mM NaCl, 50 mM Tris-HCl (pH 7.5), 5 mM MgCl_2_, 5 mM ATP, 2 mM EGTA, 1 mM dithiothreitol, 1 mg histone, and 8 μg c-Src at 37°C overnight as described [23]. Tyrosine-phosphorylated CaM, free of non-phosphorylated CaM, was obtained by affinity-purification using an immobilized anti-phospho-tyrosine antibody as previously described [24].

### Phosphodiesterase assay

The CaM-dependent cyclic nucleotide PDE1 was assayed as previously described [24] at 37°C for 15 min in 250 μl of a medium containing 50 mM imidazole-HCl (pH 7.5), 10 mM Hepes-NaOH (pH 7.4), 0.2 M NaCl, 0.4 mM β-mercaptoethanol, 5 mM MgCl_2_, 0.4 mM EGTA, 0.5 mM CaCl_2_ (0.1 mM free Ca^2+^), 2.5 mM cAMP, 0.01 units of cyclic nucleotide PDE1, 2 units of 5’-nucleotidase, and the concentrations of the different CaM species indicated in the legend to the figures. One unit of cyclic nucleotide PDE1 transforms 1 μmol of cAMP to AMP per min at 30°C and pH 7.5. One unit of 5’-nucleotidase releases 1 μmol of inorganic phosphate from AMP per min at 37°C and pH 9. The inorganic phosphate released from AMP was determined colorimetrically based in the method of Fiske and Subbarow [25].

### Nitric oxide synthase assay

Recombinant eNOS was assayed determining the accumulation of nitrite as by-product of NO oxidation using a nitrate reductase/colorimetric assay kit (Oxford Biomedical Research, UK) exogenously supplemented with 25 μM FAD, 25 μM FMN, 12 μM tetrahydrobiopterin and the concentrations of the different CaM species indicated in the legends to the figures following the recommendation of the supplier. One unit of eNOS produces 1 nmol/min of NO from arginine at 37°C and pH 7.4.

### Determination of free Ca^2+^ concentrations

The concentrations of free Ca^2+^ in the PDE1 and eNOS assay systems were determined using established algorithms of the Maxchelator program. This program is freely available at http://maxchelator.stamford.edu.

### Statistical analysis

The paired Student’s t test was performed using the Microsoft Excel (Microsoft Co., Redmon, WA) and GraphPad Prism (GraphPad Software Inc., La Jolla, CA) software programs. Data were expressed as the mean μ SEM and differences were considered significant at p ≤ 0.05 as indicated in the legends to the figures.

## Results

### Physicochemical characterization of the phospho-(Y)-mimetic CaM mutants

**Fig 1:**
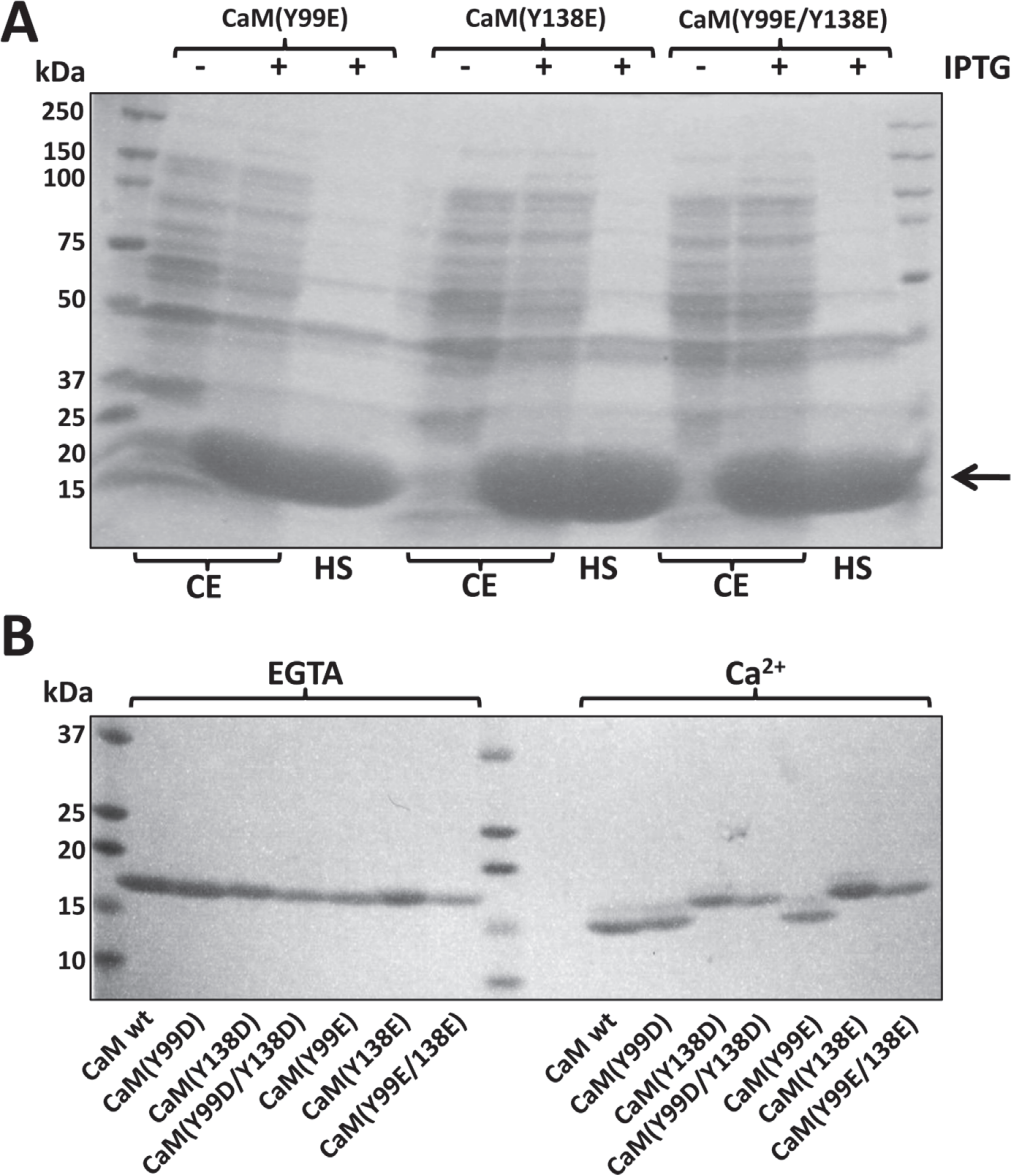
Expression and purification of the different CaM species. (*A*) The figure shows the pattern of proteins of the bacterial cell extract (CE) and the corresponding heated supernatant (HS) from *E*. *coli* BL21(DE3)pLysS transformed with plasmids encoding CaM(Y99E), CaM(Y138E) and CaM(Y99E/Y138E) in the absence (-) and presence (+) of 0.5 mM IPTG for 4 h as described in Materials and Methods. The arrow points to the band of CaM induced by IPTG. (*B*) The different recombinant CaM species (≈ 1–2 μg) purified as described in Materials and Methods were separated by SDS-PAGE in the presence of 5 mM EGTA or 1 mM CaCl_2_ (*Ca* ^*2+*^) in the loading buffer, to observe the Ca^2+^-induced mobility shift.

The expression of wild type and the different CaM mutants in *E*. *coli* BL21(DE3)pLysS was very efficient and their purification yielded an average ± SEM (n = 12) of 13 ± 2 mg CaM per 500 ml of bacterial culture. Fig 1A shows an example of the expression of the mutants CaM(Y99E), CaM(Y138E) and CaM(Y99E/Y138E) induced by IPTG addition, and the material obtained in the supernatant after heating the extracted proteins at 95°C for 5 min. It can be seen that CaM is the majoritarian heat-resistant protein. After Ca^2+^-dependent phenyl-Sepharose chromatography all purified CaM species were homogeneous in SDS-PAGE (Fig 1B). The CaM variants presented a single band (≈ 18 kDa) when electrophoretically separated in the presence of EGTA. In the presence of Ca^2+^, however, wild type CaM and both CaM(Y99D) and CaM(Y99E) mutants presented the typical Ca^2+^-induced electrophoretic mobility shift [18], yielding a major band at ≈ 15 kDa and a minor one with lower mobility. The electrophoretic mobility shift was less apparent in the single mutants CaM(Y138D) and CaM(Y138E), and in the double mutants CaM(Y99D/Y138D) and CaM(Y99E/Y138E) (Fig 1B).

**Fig 2:**
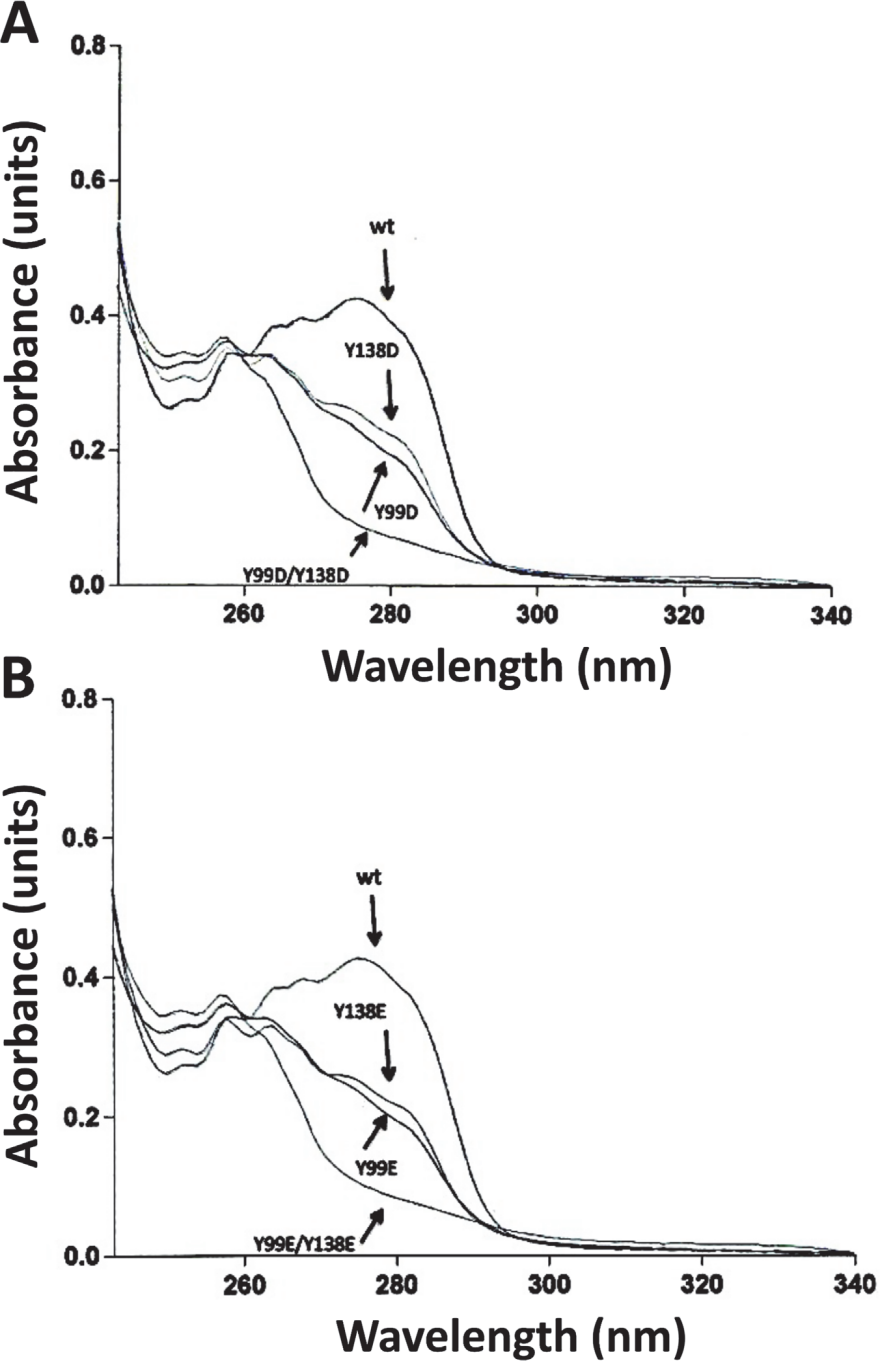
Absorption spectra of the different CaM species. The plots show UV-light absorption spectra of recombinant wild type (*wt*) and the indicated Y/D (*panel A*) and Y/E (*panel B*) CaM mutants (2 mg/ml) purified as described in Materials and Methods and dialyzed against 20 mM Tris-HCl (pH 7.5).

Fig 2 shows the UV absorption spectra (240–340 nm) of wild type CaM and the single and double Y/D (*panel A*) and Y/E (*panel B*) CaM mutants. Wild type CaM showed the characteristic absorption peaks at 252, 258, 265, 269 and 276 nm [26], the latter due to the presence of tyrosine residues. As expected, the single Y/D and Y/E CaM mutants presented a drastic reduction of the 276 nm peak, while this peak totally disappeared in the double mutants CaM(Y99D/Y138D) and CaM(Y99E/Y138E). The Y99D and Y99E substitutions yielded a slightly higher decrease in the 270–285 nm region of the absorption spectrum than identical substitutions at Y138, unveiling a higher contribution of Y99 to the spectrum. No significant spectral differences between the respective Y/D and Y/E CaM mutants were found.

**Fig 3:**
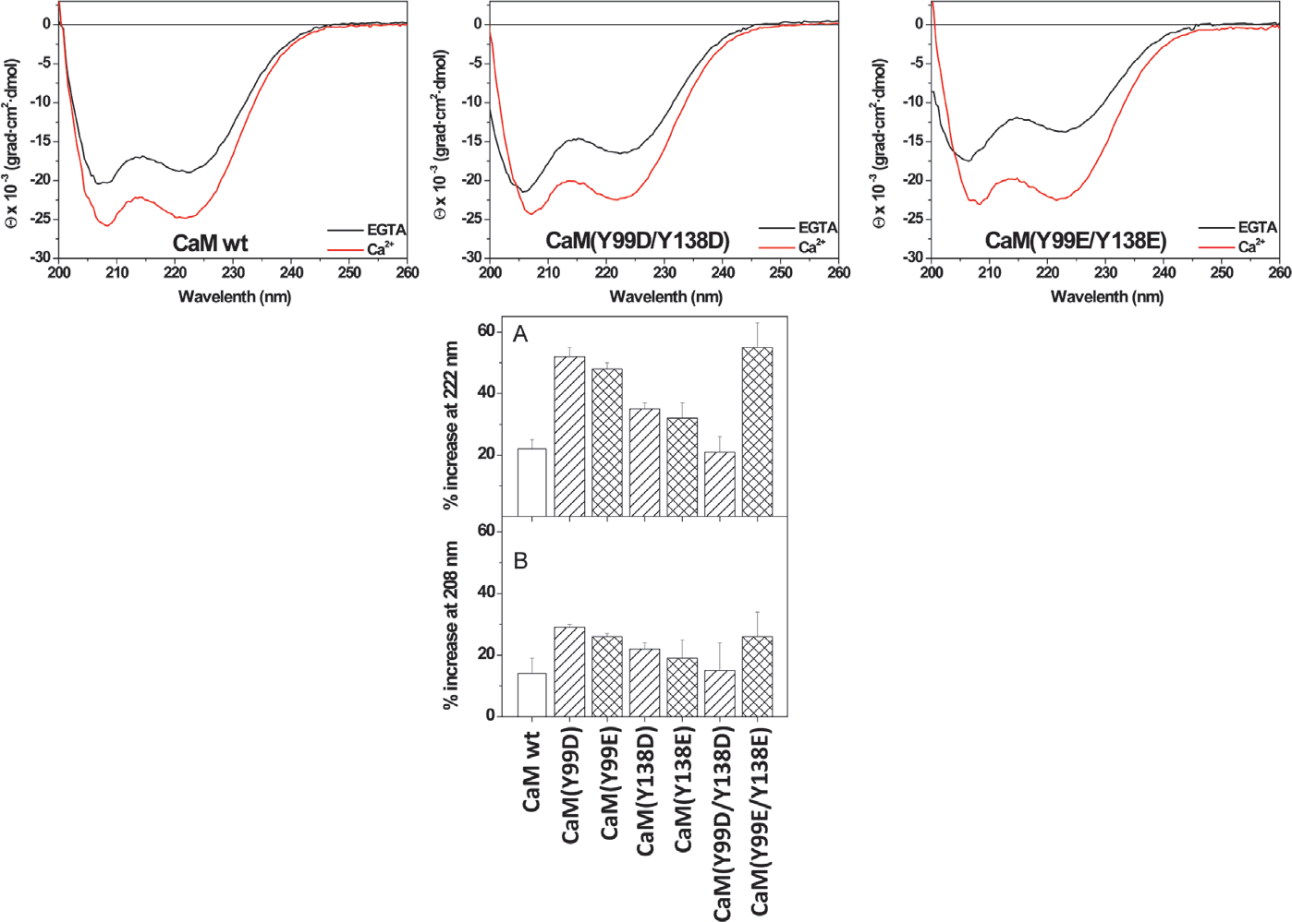
Ca^2+^-induced changes in the far-UV circular dichroism spectra of the different CaM species. (*Top panels*) Far-UV CD spectra for wild type (*wt*) and the double Y/D and Y/E CaM mutants (118 μM) were recorded *at 20°C in* 20 mM Tris-HCl (pH 7.5), 0.1 M KCl, containing 1 mM EGTA (black lines) or 1 mM CaCl_2_ (red lines). (Bottom panels) The mean ± SEM (n = 3) increase in ellipticity in the far-UV circular dichroism signals at 222 nm (*A*) and 208 nm (*B*) of the indicated CaM species is represented as percentage taking as 100% the ellipticity of each CaM species in the presence of EGTA. Measurements were carried out at 20°C in 20 mM Tris-HCl (pH 7.5), 0.1 M KCl, containing 1 mM EGTA or 1 mM CaCl_2_, respectively. The concentration of CaM was 12 μM (*A*) and 118 μM (*B*).

Circular dichroism (CD) is a very useful tool to study conformational features of proteins in solution [27]. In the absence of Ca^2+^ (presence of EGTA), the far-UV (200–260 nm) CD spectra of wild type and double Y/D and Y/E CaM mutants showed the characteristic negative maxima at 207/208 and 222 nm (Fig 3, *top panels*), typical of proteins with a high proportion of α-helical content. The same was observed for the single mutants (*not shown*). However, the ratio of molar ellipticity at 208 and 222 nm, a sensitive gauge of possible alterations in interactions between neighboring helices [19, 28], was slightly higher for the mutants (*not shown*), pointing to subtle differences in helix packing compared to wild type CaM. For all the samples, there was a significant, mutant-specific, deepening of the far-UV signals in the presence of Ca^2+^ that may reflect a differential increase in α-helical content and/or reorientation of existing α -helices [29, 30]. Also, both CaM(Y99D/Y138D) and CaM(Y99E/Y138) mutants presented a slight blue-shift of the 208 nm minimum in the absence of Ca^2+^ (presence of EGTA) as compared to wild type CaM.

As shown in Fig 3 (*panel A*), the ellipticity at 222 nm increased in the presence of Ca^2+^ 22 ± 3% for wild type CaM, while for the Y138D and Y138E mutants the increase was of 32–35%, and for the Y99D, Y99E, and double Y/E mutants it reached values of 50% and above, the only exception being the CaM(Y99D/Y138D) mutant where no significant change was observed. Interestingly, the magnitude of the increase in ellipticity at 222 nm is directly correlated to the value of the Θ_208_/Θ_222_ ratio. On the other hand, percentage increases in ellipticity at 208 nm and variations among CaM species were noticeably smaller, going from 14% to 29% (Fig 3, *panel B*). As a result, no significant differences in the Θ_208_/Θ_222_ ratio were observed in the presence of calcium, again with the only exception of the CaM(Y99D/Y138D) mutant (*not shown*), revealing a similar overall structure of the holo forms. Above all, far-UV data clearly show that all the single and double Y/D and Y/E CaM mutants bind Ca^2+^.

**Fig 4:**
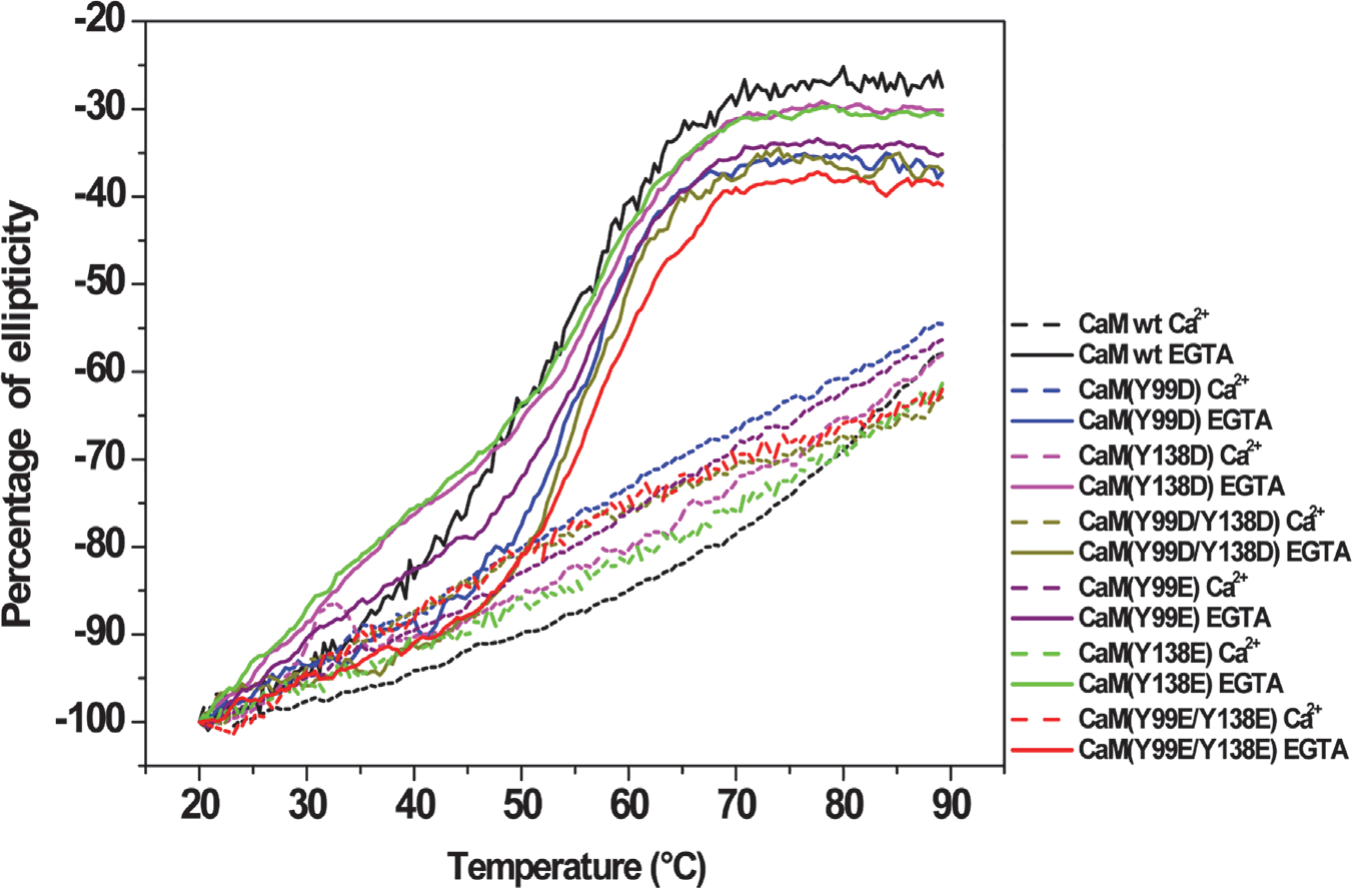
Thermal stability of the different CaM species in the absence and presence of Ca^2+^. The plots present the decrease in the CD signal at 222 nm, expressed as percentage of the ellipticity at 20°C, for wild type (*wt*) and the single and double Y/D and Y/E CaM mutants (12 μM) in 20 mM Tris-HCl (pH 7.5), 0.1 M KCl, in the presence of 1 mM EGTA (*solid lines*) and 1 mM CaCl_2_ (*dashed lines*) as described in Materials and Methods.

It is known that Ca^2+^ binding induces a remarkable increase in the thermal stability of wild type CaM [29, 31]. In order to examine whether the same is true for the single and double Y/D and Y/E mutants, we carried out thermal denaturation experiments by measuring changes in ellipticity at 222 nm at increasing temperature. Fig 4 shows a quasi-sigmoidal decrease in ellipticity of wild type and all CaM mutants in the absence of Ca^2+^ (presence of EGTA), indicating the occurrence of at least one intermediate state, as previously described for wild type CaM [19, 29]. In the case of the CaM(Y138D) and CaM(Y138E) mutants, the temperature-dependent unfolding was clearly biphasic, showing a notable deviation of sigmoidicity at temperatures below 50°C. Of note, the shape of the CaM(Y138D/E) denaturation curves is remarkably similar to that reported for a CaM Y138Q mutant [19]. Although fitting the data to a two- or three-state model did not yield acceptable clear-cut information, it is clear from the denaturation profiles that a major thermal unfolding for the different CaM species occurred at ≈ 50–55°C, all of them being completely unfolded above 70°C. In addition, lower temperature transitions and/or discontinuities, also reported for wild type CaM [31–34] were observed in the ≈ 20–40°C region, both in the absence and presence of Ca^2+^. The binding of Ca^2+^, however, had a profound impact on the stability of wild type CaM and the mutants above 40°C. Thus, the major unfolding transition occurring at ≈ 50–55°C in the presence of EGTA was not observed when Ca^2+^ is present, and more than 54% of the initial ellipticity signal was still preserved at 90°C (Fig 4), confirming that Ca^2+^ binding to the single and double Y/D and Y/E CaM mutants also causes remarkable thermal stabilization.

**Fig 5:**
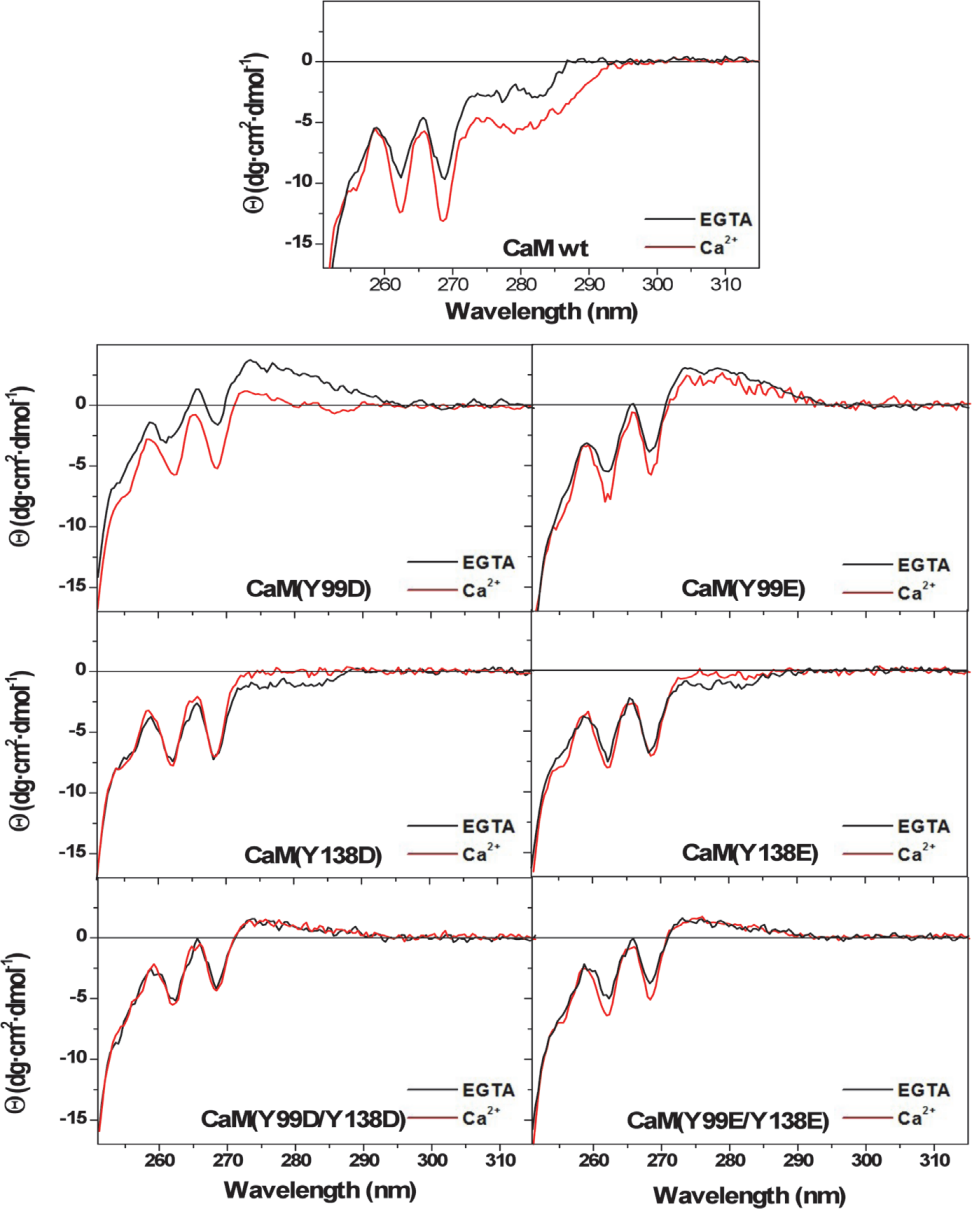
Ca^2+^-induced changes in the near-UV circular dichroism spectra of the different CaM species. Near-UV CD spectra for wild type (*wt*) and the single and double Y/D and Y/E CaM mutants (118 μM) were recorded *at 20°C in* 20 mM Tris-HCl (pH 7.5), 0.1 M KCl, containing 1 mM EGTA (black lines) or 1 mM CaCl_2_ (red lines). Ellipticity values were normalized per mole of residue.

Ca^2+^-binding also induces noticeable changes in the near-UV spectrum of wild type CaM [19, 26, 32], which is sensitive to the environment of phenylalanines and the two tyrosine residues in the molecule (please, notice that CaM does not contain tryptophan residues). As shown in Fig 5, in the presence of EGTA the spectrum is characterized by two well-defined negative bands at 262 and 269 nm, attributed to phenylalanine residues, and a broad negative region centered at 280 nm arising from the tyrosine residues. Addition of Ca^2+^ results in an increase of these ellipticity signals, what has been interpreted as a change in the environment of both phenylalanine and tyrosine residues. Having confirmed by far-UV CD and thermal denaturation experiments that all single and double Y/D and Y/E mutants bind Ca^2+^, we also examined the near-UV CD spectra of their apo and holo forms. In the absence of Ca^2+^ (presence of EGTA), the ellipticity around 280 nm of the mutants diminished noticeably or even became positive (Fig 5B–5D), confirming a predominant contribution of the two tyrosine residues to this region. In addition, there was a significant decrease in the intensity of the bands at 262 and 269 nm compared to wild type CaM, pointing to a partial contribution of both tyrosine residues to these signals. However, addition of Ca^2+^ induced different changes in the spectra depending on the mutant. While the Y99D and Y99E mutants showed a small decrease in positive ellipticity at 280 nm, for the respective Y138 mutants the ellipticity became almost zero (Fig 5). Differences related to the 262/269 nm bands were even more apparent. Here, there was a manifest increase in ellipticity for the Y99 mutants upon Ca^2+^ binding, whereas little or no change was observed for the Y138 and double mutants (Fig 5), clearly revealing that the Ca^2+^-induced alterations observed for wild type CaM in this region are mostly due to changes in the environment of Y138. This conclusion fully explains the small effect of Ca^2+^ on the near-UV CD spectrum of the separate CaM N-terminal half (residues 1–77) reported by Martin and Bayley [32], a result considered surprising by the authors in view of the similar distribution of phenylalanine residues in the N- and C-terminal halves of the protein. Indeed, the spectra with/without Ca^2+^ of the Y138 and double mutants (Fig 5) are very similar to those published for the N-terminal half [32].

**Fig 6:**
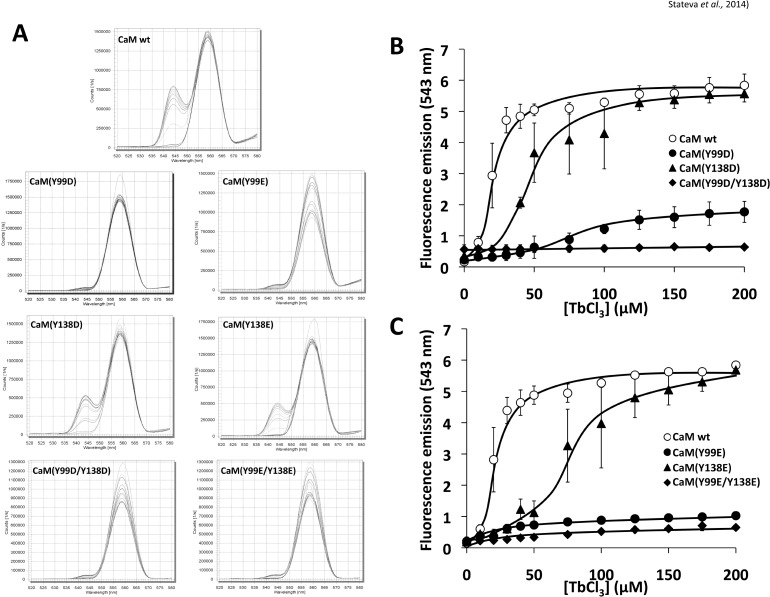
Tb^3+^-induced fluorescence emission spectra of the different CaM species. (*A*) The fluorescence emission spectra (520–580 nm) of wild type (*wt*) CaM and the indicated CaM mutants (10 μM) was recorded in a buffer containing 10 mM Pipes-HCl (pH 6.5) and 100 mM KCl in the absence and presence of increasing concentrations of TbCl_3_ (10–200 μM) as described in Materials and Methods. (*B*, *C*) The plots present the emission fluorescence at 543 nm of wild type (*wt*) CaM and the indicated CaM mutants (10 μM) at increasing concentrations of TbCl_3_ in the conditions described in *A*.

We also studied the fluorescence energy transfer from excited tyrosine residues to Tb^3+^, a surrogate ion that binds CaM at the Ca^2+^-binding sites, in wild type CaM and the different CaM mutants by measuring the fluorescence emitted by Tb^3+^ in the 535–550 nm region upon exciting at 280 nm the tyrosine residues (if present) located at sites III and IV of CaM. The spectrum obtained for wild type CaM presented a distinctive emission peak at 543 nm upon addition of increasing concentrations of Tb^3+^ to the medium (Fig 6A). Interestingly, the absence of Y99 induced an almost complete loss of the Tb^3+^ emission peak at 543 nm, while the absence of Y138 did not (Fig 6A). A similar behavior was observed for the Y/D and Y/E mutants. The Tb^3+^ titration curves shown in Fig 6B and 6C demonstrated that the apparent binding affinity of the lanthanide ion to wild type CaM was slightly higher (K’_d_ ≈ 20 μM) than for the CaM(Y138D) or CaM(Y138E) mutants (K’_d_ ≈ 50 and 75 μM, respectively). Only trace fluorescence emission of Tb^3+^ was detected in the CaM(Y99D) or CaM(Y99E) mutants, and no significant fluorescence was detected in the double Y/D or Y/E mutants as expected.

### Biological activity of the phospho-(Y)-mimetic CaM mutants

**Fig 7:**
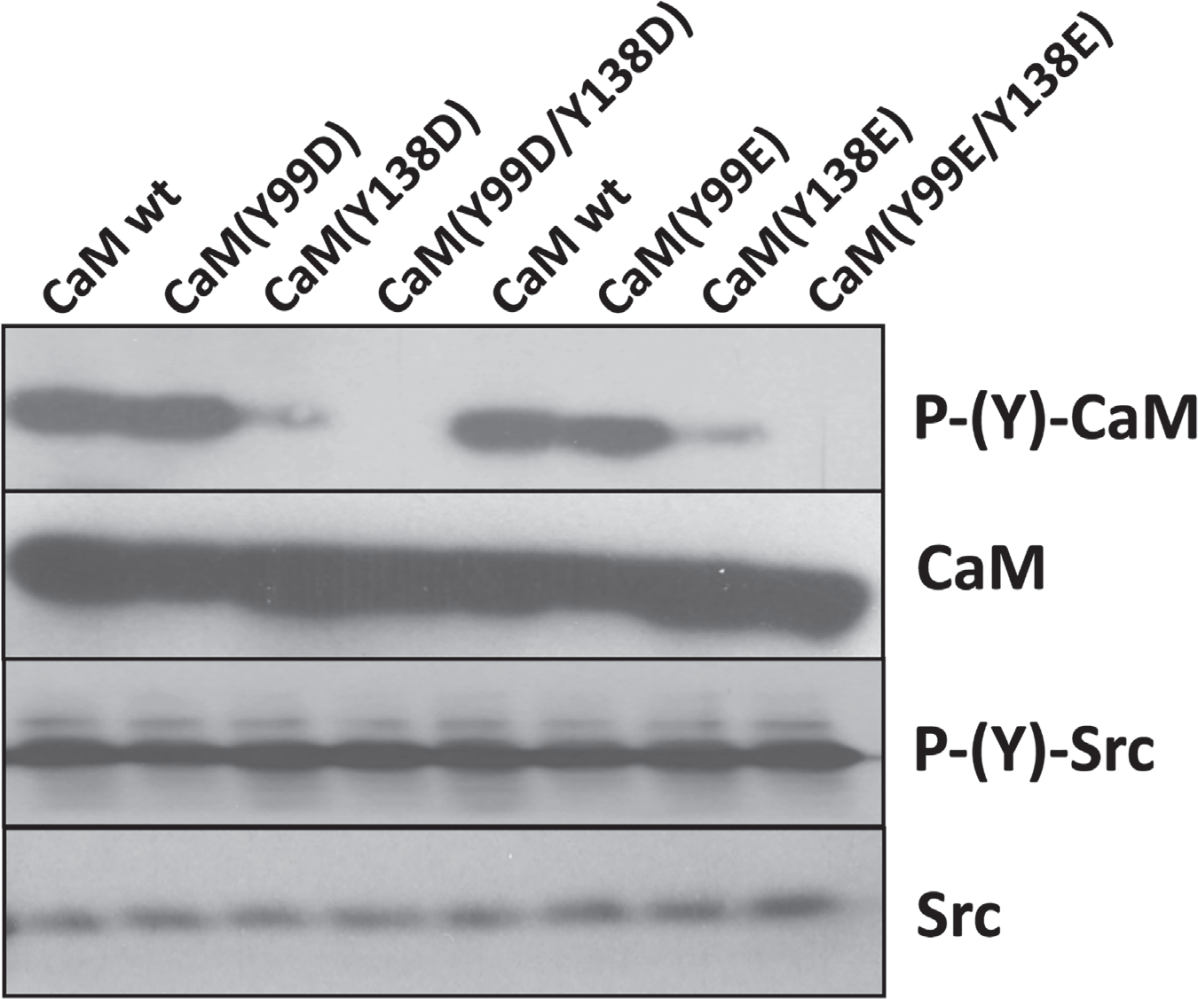
Phosphorylation of different CaM species by c-Src. The different CaM species (2 μg) were assayed for phosphorylation by recombinant c-Src as described in Materials and Methods. The samples were probed with an anti-phospho-tyrosine antibody to detect the tyrosine-phosphorylated CaM species (*P-(Y)-CaM*) and auto-phosphorylated c-Src (*P-(Y)-Src*). The membranes were striped and reprobed with anti-CaM and anti-Src antibodies as loading controls.

We first tested the capacity of the non-receptor tyrosine kinase c-Src to phosphorylate the different CaM species. Fig 7 shows that both CaM(Y99D) and CaM(Y99E) were phosphorylated *in vitro* by recombinant c-Src with similar efficiency than wild type CaM. However, the CaM(Y138D) and CaM(Y138E) mutants were phosphorylated with far lower efficiency than wild type CaM. As expected, no phosphorylation of the double mutants CaM(Y99D/Y138D) and CaM(Y99E/Y138E) was detected.

We next tested two CaM-dependent enzymes, cyclic nucleotide PDE1 and eNOS, to assess whether the different Y/D and Y/E CaM mutants have biological activity, and whether they present any differential effects on the target enzyme as compared to wild type CaM mimicking the action of phospho-(Y)-CaM.

**Fig 8:**
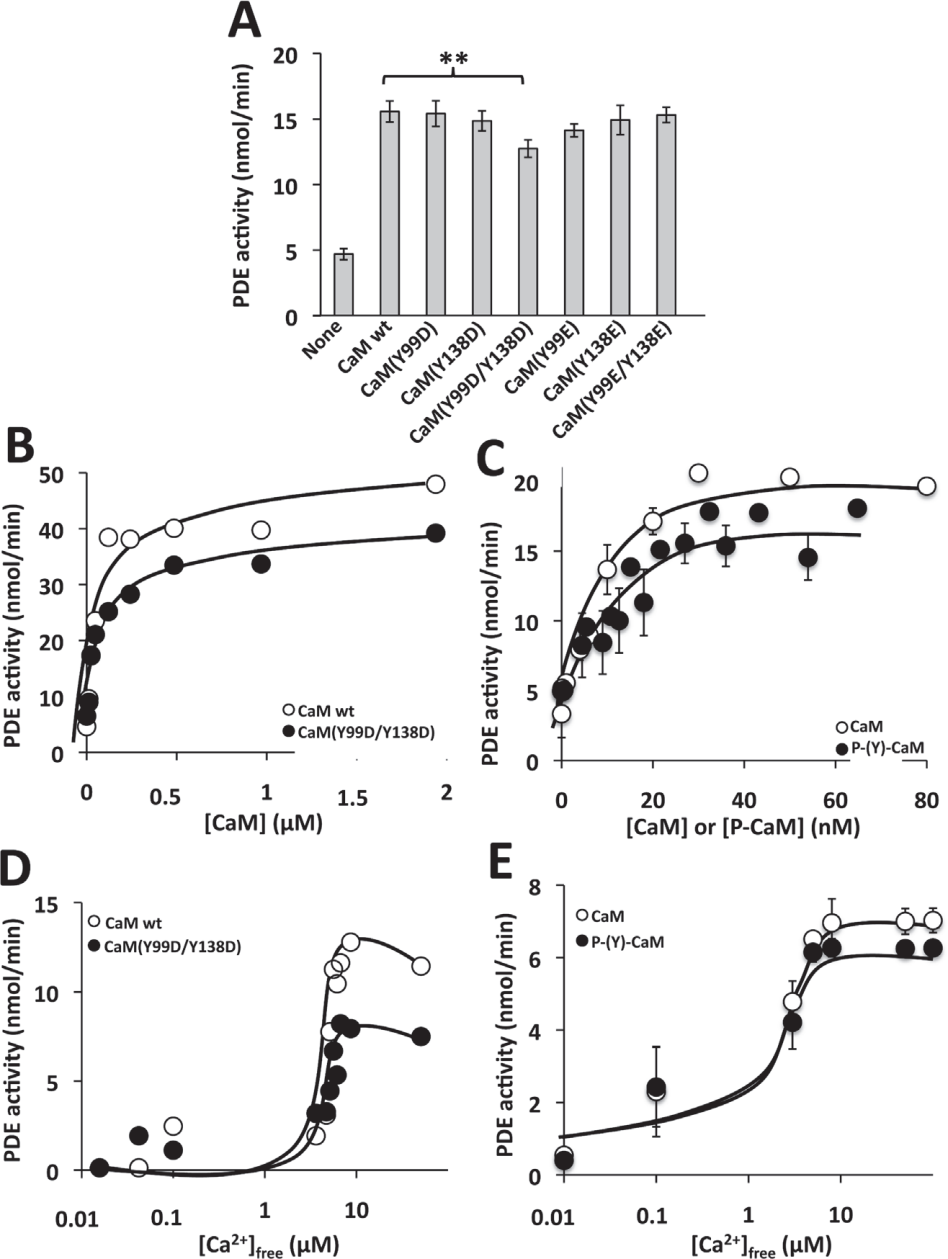
Effect of different CaM species on the activity of PDE1. (*A*) The cyclic nucleotide PDE1 activity was assayed in the absence (*None*) and presence of the indicated CaM species (1.9 μM) in the presence of 100 μM free Ca^2+^ as described in Materials and Methods. The plot presents the average ± SEM of triplicate samples from three separate experiments (** p = 0.02 using the Student’s t test). (*B*) The plots present the PDE1 activity assayed as in *A* but using increasing concentrations of the indicated CaM species. (*C*) The plot presents the average ± SEM activity of PDE1 of three independent experiments assayed at increasing concentrations of non-phosphorylated CaM (*open symbols*) or phospho-(Y)-CaM (*filled symbols*) prepared as described in Materials and Methods. (*D*) The plot presents the PDE1 activity assayed as in *A* in the presence of the indicated CaM species (1.9 μM) at increasing concentrations of free Ca^2+^ using an EGTA/Ca^2+^ buffer as described in Materials and Methods. (*E*) The plot presents the average ± range activity of PDE1 assayed in the presence of non-phosphorylated CaM (*open symbols*) and phospho-(Y)-CaM (*filled symbols*) (0.97 μM) of two independent experiments assayed at increasing concentrations of free Ca^2+^ using an EGTA/Ca^2+^ buffer as described in Materials and Methods.

Fig 8A shows that all the Y/D and Y/E CaM mutants were able to activate PDE1 in the presence of Ca^2+^ as it does wild type CaM. However, the CaM-dependent activity attained with CaM(Y99D/Y138D) was consistently lower (≈ 20%) than with the other CaM species, including the CaM(Y99E/Y138E) mutant. PDE1 assays performed at increasing concentrations of CaM(Y99D/Y138D), as compared to wild type CaM, showed no significant differences in the K_act_ (≈ 5–10 nM), but a small decrease (≈ 20%) in the V_max_ of the enzyme was noticeable (Fig 8B). The lower activatory effect of this mutant was of the same magnitude that the one observed when purified bovine brain phospho-(Y)-CaM (phosphorylated by c-Src and free of non-phosphorylated CaM) was assayed on the PDE1 (Fig 8C). When the assays were performed at increasing concentrations of free Ca^2+^ no significant changes in the apparent affinity for free Ca^2+^ (≈ 5 μM) between wild type and the double Y/D CaM mutant was noticed. And again a decrease (≈ 20–30%) in the CaM-dependent activity of PDE1 at saturating concentrations of free Ca^2+^ was observed with CaM(Y99D/Y138D), as compared to wild type CaM (Fig 8D). Moreover, assays performed at increasing concentrations of free Ca^2+^ in the presence of no-phosphorylated CaM and phospho-(Y)-CaM showed no significant differences in the apparent affinity for free Ca^2+^ (Fig 8E).

**Fig 9:**
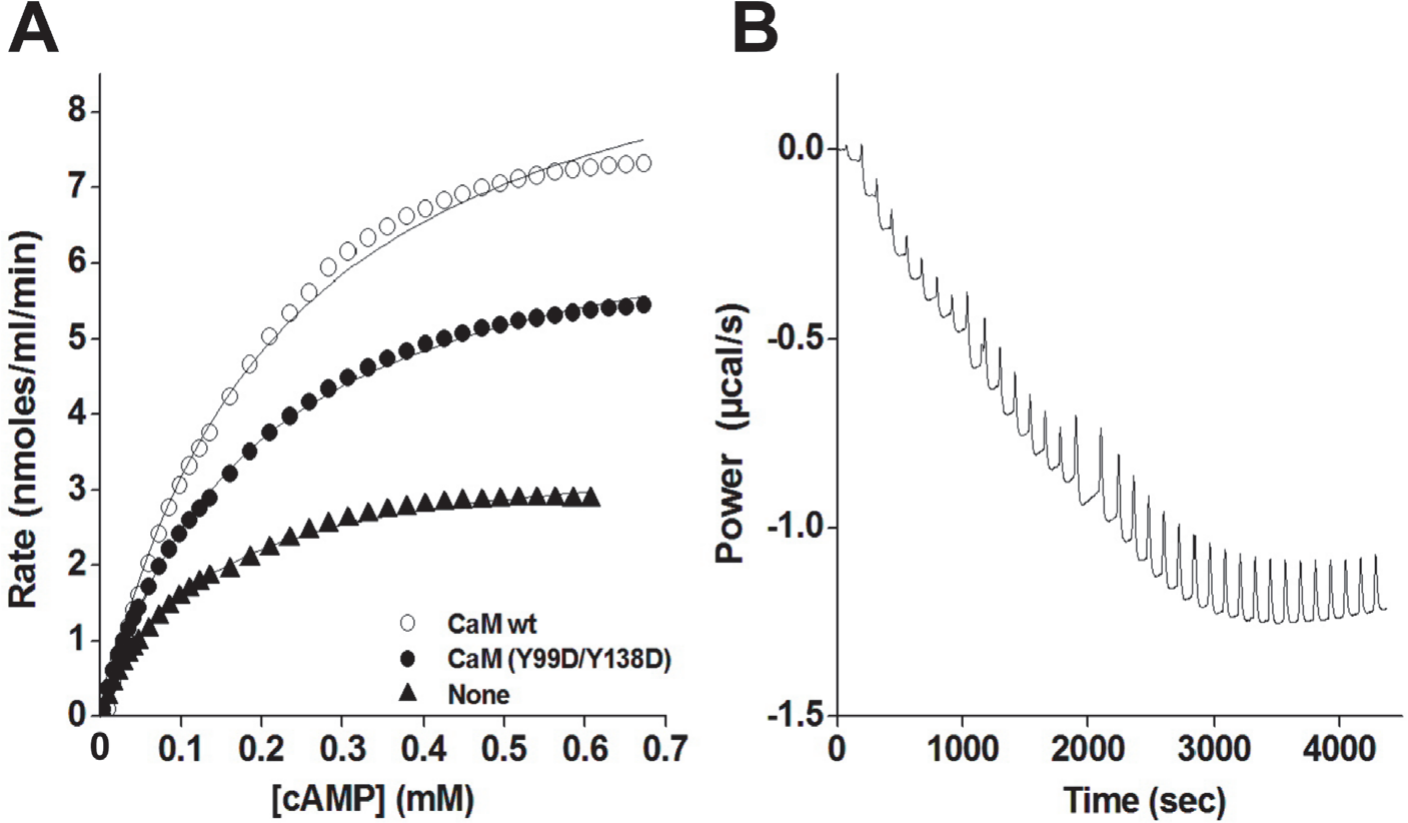
Effect of different CaM species on the kinetics of PDE1 determined by isothermal titration calorimetry. (*A*) The plot presents the PDE1 activity (normalized for 0.04 units of enzyme) at increasing concentrations of cAMP in the absence (*filled triangles*) and presence of wild type CaM (*open circles*) or CaM(Y99D/Y138D) (*filled circles*) (3.8 μM) in a buffer containing 50 mM imidazol-HCl (pH 7.5), 200 mM NaCl, 5 mM MgCl_2_, 0.4 mM EGTA, and 0.5 mM CaCl_2_ using ITC as described in Materials and Methods setting the micro-calorimeter chamber 37°C. (*B*) The trace corresponds to a typical experiment showing the rate of heat produced by PDE1 over time after injections of different pulses of cAMP from which the activity of the enzyme can be derived as described in Materials and Methods. This particular trace corresponds to an experiment performed in the absence of CaM using 0.04 units of PDE1. Similar traces were obtained in other conditions tested.

We also determined additional kinetics parameters of PDE1 by ITC using increasing concentrations of cAMP in the absence and presence of wild type CaM and CaM(Y99D/Y138D). Fig 9 shows a typical experiment where the presence of wild type CaM greatly enhanced PDE1 activity, while CaM(Y99D/Y138D) had a lesser effect. We determined in a series of similar experiments that the PDE1 activity increased 2.61 ± 0.22 and 1.94 ± 0.09 folds (n = 3, p < 0.05) upon addition of wild type CaM and CaM(Y99D/Y138D), respectively. Moreover, the V_max_ was determined to be 7.4 ± 2.8 and 5.5 ± 2.3 nmoles/s (n = 3, p < 0.05) in the presence of wild type CaM and CaM(Y99D/Y138D), respectively, while the K_m_ of the enzyme for cAMP was the same (221 ± 27 μM) in the presence of both CaM species.

**Fig 10:**
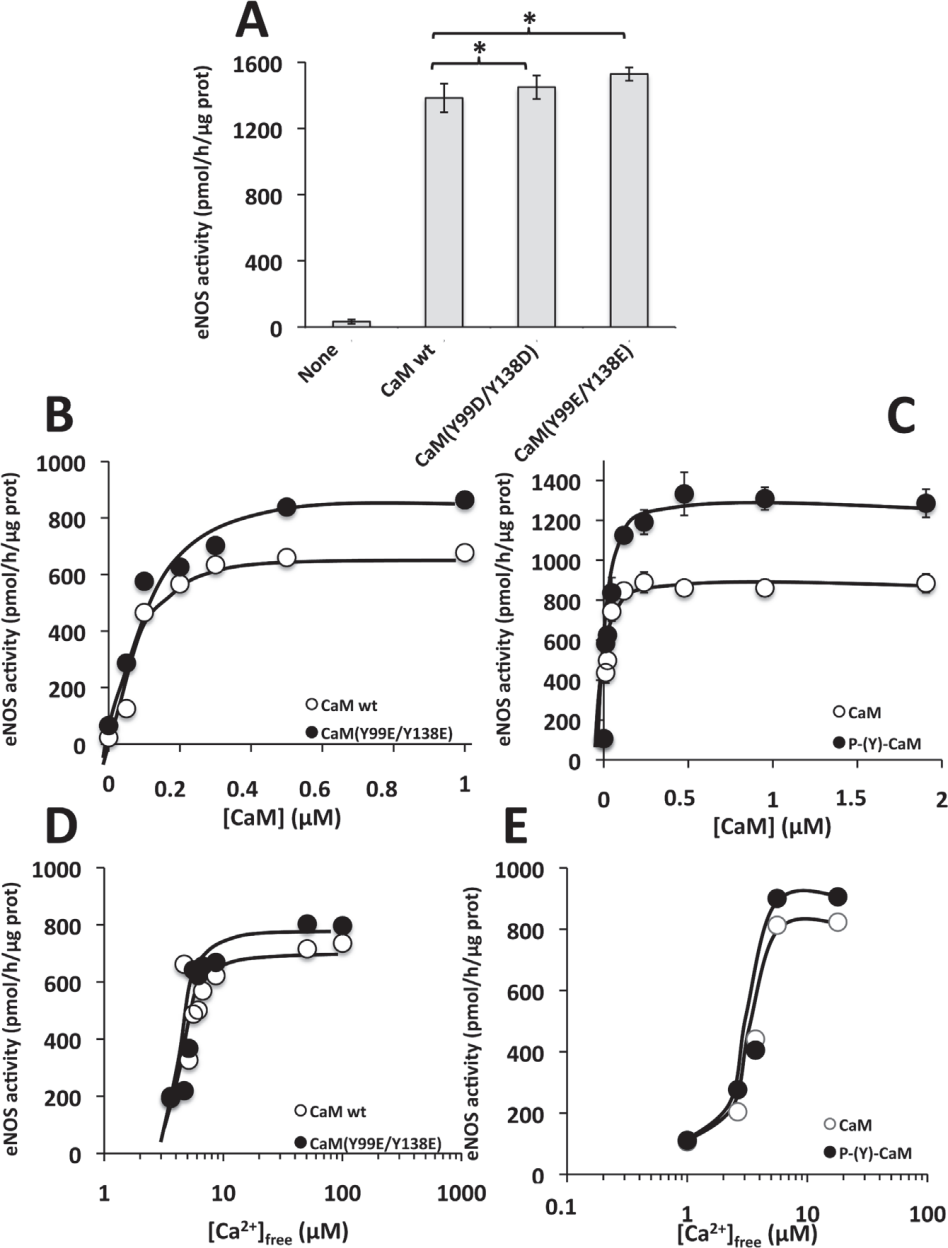
Effect of different CaM species on the activity of eNOS. (*A*) The activity of eNOS was assayed in the absence (*None*) and presence of the indicated CaM species (4.7 μM) in the presence of 1 mM free Ca^2+^ as described in Materials and Methods. The plot presents the average ± SEM of triplicate samples from three separate experiments (* p ≤ 0.05 using the Student’s t test). (*B*) The plot presents the eNOS activity assayed as in *A* but using increasing concentrations of the indicated CaM species. (*C*) The plot presents the average ± range activity of eNOS of two independent experiments assayed at increasing concentrations of non-phosphorylated CaM (*open symbols*) or phospho-(Y)-CaM (*filled symbols*) prepared as described in Materials and Methods. (*D*) The plot presents the eNOS activity assayed as in *A* in the presence of the indicated CaM species (4.7 μM) at increasing concentrations of free Ca^2+^ using an EGTA/Ca^2+^ buffer as described in Materials and Methods. (*E*) The plot presents the activity of eNOS assayed as in *A* in the presence of non-phosphorylated CaM (*open symbol*) and phospho-(Y)-CaM (*filled symbols*) (0.6 μM) assayed at increasing concentrations of free Ca^2+^ using an EGTA/Ca^2+^ buffer as described in Materials and Methods.

We finally tested the comparative action of wild type CaM, CaM(Y99D/Y138D) and CaM(Y99E/Y138E) on the activation of recombinant eNOS. Fig 10A shows that both CaM mutants strongly activate eNOS in assays performed in the presence of Ca^2+^, and this activation was slightly more efficient than when wild type CaM was used. When eNOS was assayed at increasing concentrations of the different CaM species (Fig 10B), no significant differences in the K_act_ (≈ 0.1 μM) were observed between wild type and the double Y/E CaM mutant. In contrast, a slight increment in the V_max_ (≈ 30%) with respect to wild type CaM was noticeable with CaM(Y99E/Y138E). Similarly, phospho-(Y)-CaM increased the activity of eNOS more efficiently (≈ 30%) than non-phosphorylated CaM (Fig 10C). When the assays were performed at increasing concentrations of free Ca^2+^ no significant changes in the apparent affinity for free Ca^2+^ (≈ 4–6 μM) when compared wild type CaM and the double Y/E CaM mutant (Fig 10D). Moreover, no differences in the apparent affinity for free Ca^2+^ for non-phosphorylated CaM and phospho-(Y)-CaM were detected (Fig 10E).

## Discussion

In contrast to the homogeneous electrophoretic migration (≈ 18 kDa) of the single or double Y/D and Y/E CaM mutants in the absence of Ca^2+^ (presence of EGTA), and the near absence of Ca^2+^-induced electrophoretic mobility shift of the single or double CaM mutants where Y138 was substituted by acidic residues (D or E) observed in this work, the non-phosphorylatable Y/F CaM mutants previously described presented a different behavior [22]. Thus, the CaM(Y138F) and CaM(Y99F/Y138F) mutants presented in the absence of Ca^2+^ (presence of EGTA) higher electrophoretic mobility than wild type CaM and CaM(Y99F), and even higher Ca^2+^-induced mobility shift [22]. This suggests that the substitution of Y138 either by an acidic or a hydrophobic residue exerts distinct effects on the conformation of CaM. Our results are in agreement with previous findings in which a differential behavior was observed when Y138 or Y99 were substituted by diverse amino acids, suggesting that mutation of Y138 disrupts the structural coupling between the N and C lobular halves of CaM potentially mimicking the collapse that CaM undergoes when binding to target proteins [19, 35]. The central position of Y138 in the Ca^2+^-induced conformational change of CaM is also evidenced by the modification of the chiroptical properties of this residue, here revealed to be the culprit for the well-known changes in the near-UV CD spectrum of CaM. Of note, the spectrum of Ca^2+^-bound CaM is very similar to that of *Drosophila melanogaster* calmodulin [36], which only contains this tyrosine residue but not that at position 99, further substantiating this conclusion.

The far-UV CD spectra of the different phosphomimetic CaM mutants as compared to wild type CaM in the absence and presence of Ca^2+^ suggest that the substitution of either one or the two tyrosine residues does not impair Ca^2+^ binding, as the presence of Ca^2+^ induces a similar conformational change in the molecules. Furthermore, the thermal stability of all the mutants is remarkably increased in the presence of Ca^2+^, showing that Ca^2+^ binding has a similar stabilizing effect on wild type and the mutant CaM species. This conclusion is also supported by the fact that all the Y/D CaM mutants required Ca^2+^ to stimulate the activities of PDE1 and eNOS, as it was the case for wild type CaM. Therefore, the absence of a significant Ca^2+^-induced electrophoretic mobility shift in the Y138D and Y138E CaM mutants is not a valid criterion to propose absence of Ca^2+^-binding capability.

The near loss of fluorescence emission by Tb^3+^ upon tyrosine excitation in the single mutant lacking Y99, but not the single mutant lacking Y138, suggests that Tb^3+^ is closer to tyrosine 99 located at the III Ca^2+^-binding site of CaM than to tyrosine 138 located at its IV Ca^2+^-binding site. This is in full agreement with the crystallographic structure of CaM, where the carbonyl group of Y99 participates in the coordination of Ca^2+^, while Y138 has a rather outward projecting conformation unable to coordinate Ca^2+^ [37].

In agreement with the distinct spatial projection and accessibility to kinases of the two tyrosine-residues of CaM, our c-Src phosphorylation experiments show that Y99 was phosphorylated with lower efficiency than Y138 by this kinase. The inward projection of Y99 within the III Ca^2+^-binding pocket as compared to a more outward projection of Y138 within the IV Ca^2+^-binding pocket [37] may explain these results. Although both tyrosine residues in CaM are phosphorylated by both c-Src and EGFR, as demonstrated using Y99F and Y138F CaM mutants [22], the substitution of the tyrosine residue at position 138 by a non-phosphorylatable amino acid somehow disturbs in part the accessibility of these kinases to Y99. This is remarkable as it was previously suggested that the phosphorylation of Y99 by several tyrosine kinases was more efficient than the phosphorylation of Y138 with the same kinases (reviewed in [10]). Thus, the phosphorylation of single-tyrosine CaM mutants may not reflect the comparative efficiency of phosphorylation of both tyrosine residues of CaM in living cells. This may be related to the fact that substituting Y138 by other amino acids disrupts the structural coupling between the N- and C-globular domains of CaM [19], as previously mentioned.

Our results also show that the single and double Y/D and Y/E CaM mutants all retain biological activity, as they were able to activate distinct CaM-binding enzymes including PDE1, eNOS, and the plasma membrane Ca^2+^-ATPase (*not shown*). Moreover, we have shown a differential action of wild type CaM and some of the phospho-(Y)-mimetic CaM mutants under study on the activity of PDE1 and eNOS.

The double mutant CaM(Y99D/Y138D), but not CaM(Y99E/Y138E), exerts a lower activatory effect on the V_max_ of the CaM-dependent cyclic nucleotide PDE1 isolated from bovine brain, as compared to wild type CaM, but it does not significantly change the apparent affinity for Ca^2+^ or the activation constant (K_act_) of CaM for the enzyme. Other studies on the effect of phospho-(Y)-CaM versus non-phosphorylated CaM on the cyclic nucleotide PDE1 of different origin yielded divergent results. Thus, both an increase [38] and a decrease [23] in the K_act_, without affecting the V_max_, has been reported for phospho-(Y)-CaM (phosphorylated by the insulin receptor) and a phospho-(Y99)-CaM species, respectively, on the PDE1 from bovine brain in comparison to non-phosphorylated CaM. In contrast, phospho-Y-CaM (phosphorylated by the insulin receptor, preferably at Y99) presented similar K_act_ and V_max_ than with non-phosphorylated CaM when assayed on the PDE1 from rat hepatocytes, although the phosphorylated form increased the IC_50_ of CaM antagonists inhibiting the PDE1 activity [39]. In radical contrast, phospho-(Y)-CaM (phosphorylated by the EGFR) inhibited by 90% the activity of the PDE1 from bovine heart [24].

These discrepancies may be due to the fact that at least eleven families of distinct cyclic nucleotide PDEs, including different isoforms of the CaM-dependent PDE1, with distinct affinities for cAMP and/or cGMP and diverse regulatory properties may coexist and/or being differentially expressed at distinct ratios in various tissues and/or cell types in diverse organisms [40]. Alternatively, distinct phospho-Y99/phospho-Y138 ratios in phospho-(Y)-CaM may account for the observed discrepancies. Nevertheless, it is likely that the negative charges of the extra aspartic acids present in CaM(Y99D/Y138D) may exert a similar action that phospho-(Y99/Y138)-CaM in some PDE1 isoform(s). Furthermore, the absence of effect of the single Y/D mutants suggests that the phosphorylation of both tyrosine residues may be necessary for the lower activatory action of phospho-(Y)-CaM on PDE1. The absence of significant effect of the CaM(Y99E/Y138E) mutant also suggests that the length of the side chain of glutamic acid, longer than the one of aspartic acid (3.39 Å and 2.56 Å, respectively, from the carboxylic to the C-α) [41], may be unfitted to interact with the PDE1 site where phospho-(Y)-CaM exerts its regulatory action on the enzyme.

We show that the mutant CaM(Y99D/Y138D), and most prominently CaM(Y99E/Y138E), slightly increase (≈ 20–30%) the V_max_ of eNOS as compared to wild type CaM. Other studies, however, have shown that the single mutant CaM(Y99E) slightly increase (16%) the activity of recombinant inducible nitric oxide synthase (iNOS), has no significant effect on neuronal nitric oxide synthase (nNOS), and significantly inhibited (40%) the activity of eNOS [42]. This suggests that the different NOS isoforms may be regulated differently and that the phosphorylation of either one or the two tyrosine residues of CaM may exert significant differential effects on its activity. Nevertheless, our results are qualitatively, but not quantitatively, in agreement with the stimulatory action that phospho-(Y99)-CaM exerts on the V_max_ of nNOS, where a 3.4-fold higher V_max_ than with non-phosphorylated CaM was reported [23]. These authors also reported a two-fold higher K_act_ of phospho-(Y99)-CaM with respect to non-phosphorylated CaM, and a 4-fold lower K_d_ for a peptide corresponding to the CaM-binding domain of nNOS [23]. The different NOS isoforms used in these studies could explain the lower phospho-mimetic capacity of CaM(Y99D/Y138D) and CaM(Y99E/Y138E) when compared to tyrosine-phosphorylated CaM.

It is intriguing that CaM(Y99D/Y138D), but not CaM(Y99E/Y138E), exerts a differential action on PDE1, as compared to wild type CaM; while in the case of eNOS this differential action was exerted by both CaM(Y99D/Y138D) and CaM(Y99E/Y138E), albeit the latter with better efficiency. This could be related to the different length of the side chain of glutamic and aspartic acids as mentioned above. Thus, although the actual reason for these distinct effects is unknown, we could speculate that each enzyme may have pockets or clefts of distinct depths were the negative charge of the carboxyl group of the acidic amino acid must adapt in a proper conformation in order to exert its differential action, and that this pocket/cleft may be deeper in PDE1 than in eNOS.

Although the phospho-(Y)-mimetic CaM mutants described in this work exert small differential effects with respect to wild type CaM on the two CaM-binding enzymes tested, the observed effects are qualitative consistent with those exerted by phospho-(Y)-CaM. Therefore, these mutants might be useful tools to study the differential regulation exerted by phospho-(Y)-CaM in a wide variety of CaM-binding proteins with and without enzymatic activity in living cells. This could be achieved by the stable expression of these phospho-(Y)-mimetic CaM mutants in a conditional CaM knockout DT40 cell line recently described, after the down-regulation of endogenous CaM by tetracycline, where the action of distinct Ca^2+^-null and other CaM mutants on the viability and proliferation capacity of these cells was tested [43].
